# Bone filler and adhesive at the same time: in-vitro analysis in a porcine fracture model

**DOI:** 10.1186/s12891-025-08773-y

**Published:** 2025-05-27

**Authors:** Stefanie Hoelscher-Doht, Nicola Zufall, Maximilian Heilig, Philipp Heilig, Martin Cornelius Jordan, Rainer Heribert Meffert, Uwe Gbureck, Lea Hüls

**Affiliations:** 1 Department for Trauma, Hand, Plastic and Reconstructive Surgery, University Hospital Würzburg, Oberdürrbacher Strasse 6, 97080 Würzburg, Germany; 2 Department of Trauma and Orthopaedic Surgery, BG Unfallklinik Frankfurt Am Main, Friedenberger Landstrasse 430, 60389 Frankfurt am Main, Germany; 3 Center of Orthopedics, Trauma Surgery and Rehabilitation Medicine, University Medicine Greifswald, Fleischmannstraße 8, 17475 Greifswald, Germany; 4 Department for Functional Materials in Medicine and Dentistry, University Hospital Würzburg, Pleicherwall 2, 97070 Würzburg, Germany

**Keywords:** Bone cement, Filler, Bone adhesive, Fracture model, Biomechanical test, Sticky

## Abstract

**Background:**

Bone defects in the context of fracture treatment or tumor surgery represent a major challenge regarding their treatment. Sticky and drillable magnesium phosphate cements could revolutionize the intraoperative reconstruction of complex fractures close to the joint due to their properties as bone adhesive and filler at the same time, enabling the technique of first reduction of the fracture fragments by bonding with the cement and then applying stabilization with screws and/or plates.

**Methods:**

Lateral split-depression fractures of the proximal tibia were generated in 27 porcine specimens, which were then randomized into 3 groups of 9 each. In group A, a new operative technique was applied by reducing the fracture using a newly formulated magnesium phosphate cement (MgP cement) and then applying stabilization by plate osteosynthesis. In the other two groups, plate osteosynthesis was performed first, as in the current standard operative procedure, followed by the injection of a bone graft substitute through a gap in the fracture area of the tibia, group B with MgP cement, group C with hydroxyapatite cement. The following parameters were determined during the cyclic testing phase of 3000 test cycles: The total displacement and the optical displacement of the lateral plateau [mm]. During load-to-failure tests, the stiffness [N/mm], the maximum load [N] and the normalized maximum load [%] were determined.

**Results:**

The results revealed a comparable stability for all groups with no significant differences in all forms of displacement, with group A demonstrating the lowest values for displacement. Maximum load was highest for group C (group B; C [*p* = 0.04]; group A; C [*p* < 0.01]), however considering normalized maximum load, no significant difference between the three groups could be found.

**Conclusions:**

This study presents a breakthrough approach using a bone cement as both a bone adhesive and a filler at the same time. The adhesive and drillable magnesium phosphate cement proved to be a versatile solution featuring a new surgical method in which the fracture was anatomically reduced using only the cement. Furthermore, with this new technique, the cement demonstrated comparable, if not slightly superior, biomechanical stability in the porcine tibial split depression fracture model compared to the current standard of surgical treatment using primary plate osteosynthesis and a commercial hydroxyapatite cement.

## Background

Bone defects in the context of fracture treatment or tumor operations are a major challenge in orthopedics and trauma surgery. Osteosynthesis alone as a general principle does not provide sufficient stabilization in both the short and long term [1, 2]. In the short term, subsidence of joint surfaces may occur, especially in areas close to the joint. In the long term, repetitive loading leads to fatigue of the stabilization with loosening and implant failure resulting in screw or plate breakage. Autologous bone grafts have disadvantages such as harvesting morbidity, size limitation and lack of primary stability, which is why bone substitutes are often used instead [3–5]. Distinctions are made between solid bone substitute materials in the form of blocks or granules as allografts (from body donors), xenografts (e.g. from bovine cancellous bone) or made of calcium phosphate. These solid bone substitutes have the disadvantage of failing to achieve relevant primary stability. In addition, the acceptance of allografts and xenografts among patients is very limited. Therefore, mineral bone substitutes based on calcium phosphate are more commonly used in clinical applications [4–8]. In contrast to solid bone substitutes, injectable materials offer the advantage of minimally invasive injection and application into the bone defect, as well as enabling more effective bone defect filling. The current standard of injectable bone graft substitutes in clinical use is represented by hydroxyapatite cements, which, in addition to adequate processing time and viscosity, exhibit sufficient primary stability, especially under axially applied forces, as has been demonstrated in clinical studies [5, 9]. As a result, these mineral bone substitutes are widely used for bone defects close to the joint, as for example at the tibial plateau or at the calcaneus, where a high stability of the bone substitute is crucial to prevent subsidence of the joint-bearing surfaces for subsequent patient outcome. The filling of the bone defect is more extensive and thus complete if the bone substitute material is injected initially, and only afterwards a stabilization by screws (with or without plate osteosynthesis) is performed [10]. However, for this approach to be effective and successful, the bone graft substitute must provide properties that allow for subsequent drilling and screwing without compromising its stability. Nevertheless, such a bone graft substitute is currently not available for clinical use but would be a great asset in patient care in the clinical setting.

Experimental magnesium phosphate cements (MgP) represent an alternative to hydroxyapatite cements [11–14]. In initial studies, injectable struvite cements showed both high primary stability and good biocompatibility with rapid degradation in various defect situations in an ovine model, accompanied by significantly improved new bone formation compared to calcium phosphates [15]. One modification of MgP involves the addition of phytic acid as a liquid component [16, 17]. Phytic acid reacts with magnesium ions to form chelate complexes and shows both strong cohesion and adhesion to bone substrates. MgP consisting of trimagnesium phosphate, magnesium oxide and phytic acid have been further developed with a focus on improving desirable clinical properties. Two cement compositions were convincing with excellent properties in the material tests and in the clinical fracture model on synthetic bone: drillability and high stability at the same time [16]. Interestingly, mineral cements with phytic acid as a liquid component also exhibit adhesive properties [17].

An adequate bone adhesive has been targeted for many years. However, all previous experimental approaches to adhesive development showed deficiencies regarding inflammatory responses, stress shielding and mechanical strength [18, 19]. Bone cements have the great advantage of already being optimally suited in their properties for application to bone. The incredible accidental finding of a sticky and drillable bone cement now makes it possible for the first time to combine the use of the cement as a bone defect filler as well as a bone adhesive.

Especially with respect to the treatment of complex fractures close to the joint, this would revolutionize the surgical techniques and possibilities of intraoperative reconstruction, for example, of the joint block via the adhesive cement and then later additional stabilization via screws. At the same time, a metaphyseal defect zone could be filled with cement. Under ideal conditions, an anatomical reduction of a multi-fragment fracture can be achieved with the adhesive cement, followed by additional stabilization via osteosynthesis. Due to the drillability of the adhesive cement, unchanged stability of the cement is guaranteed even after screw fixation.

The aim of this study was to evaluate the potential use of an adhesive and drillable MgP in a set-up as realistic as possible. For this purpose, a fracture model with a depressed component and an intra-articular fracture component had to be established first, on which the new surgical method with anatomical reduction of the fracture using adhesive cement could be verified. Subsequently, a biomechanical analysis of the stability of the new surgical method was performed in vitro. The working hypothesis was that sticky MgP can be used to anatomically reduce a fracture with a bone defect close to the joint and that this method ensures the same primary stability as the current standard of surgical treatment with primary plate osteosynthesis and a commercial hydroxyapatite cement.

## Methods

### Fracture model

**Fig. 1:**
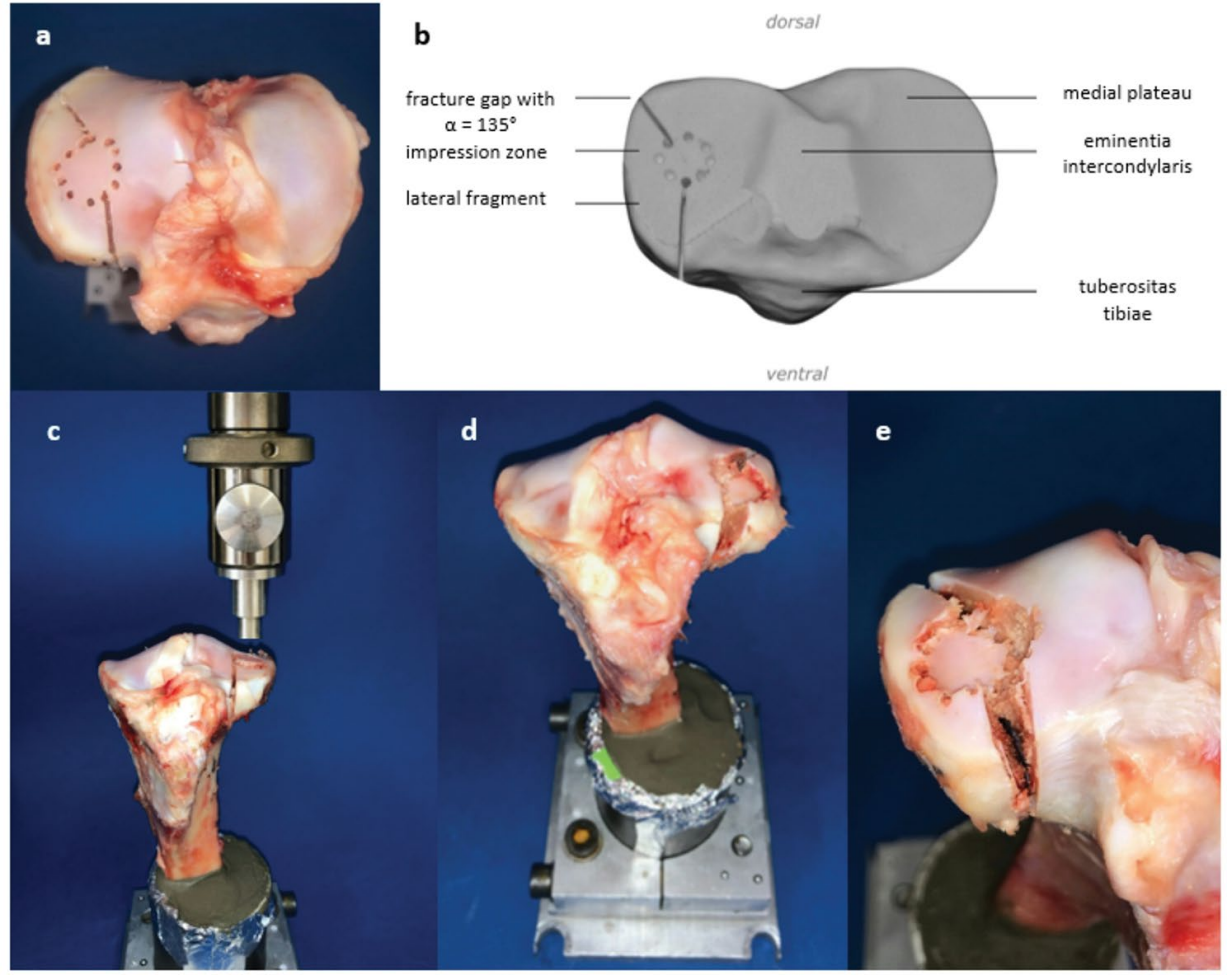
The lateral plateau during the preparation and fracture generation is shown. The drilling and osteotomies in the lateral plateau from above (**a**) are labeled in the schematic drawing (**b**) to demonstrate the exact angle α = 135°. Afterwards, the porcine tibia was placed under a stamp of the material testing machine (**c**), the split depression fracture (**d**) was generated and demonstrated with a closer view (**e**). (**d**) shows the porcine bone in the metal embedding device

A lateral split depression fracture of the proximal tibia was generated in 27 porcine tibiae (Fig. 1) from 6 months old domestic house pigs. These bones were harvested from pig lower legs that accumulate as slaughterhouse waste provided by the local butchery Hollerbach GmbH, Rimpar, Germany. The hole lower leg was initially fresh-frozen in, with physiological saline soaked, dressings. Before testing, the legs were warmed to room temperature (20°) during 24 h. The tibiae were initially dissected to separate the tibial bone from the femur, menisci, capsule, and other connective tissue. After shortening the tibiae at the distal end to a total length of 20 cm, they were placed in a metal device with a 5° valgus angulation [20]. To simulate a depression zone in the anterolateral area of the plateau, 12 × 2 mm holes with a depth of 5 mm were arranged in a circle measuring a diameter of 12 mm [21]. Two incomplete osteotomies next to the depression zone with a 135° angle to each other were used to specify the fracture line (Fig. 1a, b). After the preparations, the bone was fixed in the material testing machine Zwick Roell Z020 (ZwickRoell GmbH, Ulm, Germany) (Fig. 1c). After precise alignment of a stamp over the previously marked location, the machine simulated the split-depression fracture at a test speed of 25 mm/min (Fig. 1d, e). The depression depth was limited to 10 mm and the maximum force applied was recorded by the software testXpert III.

### Group classification and surgical technique

**Fig. 2:**
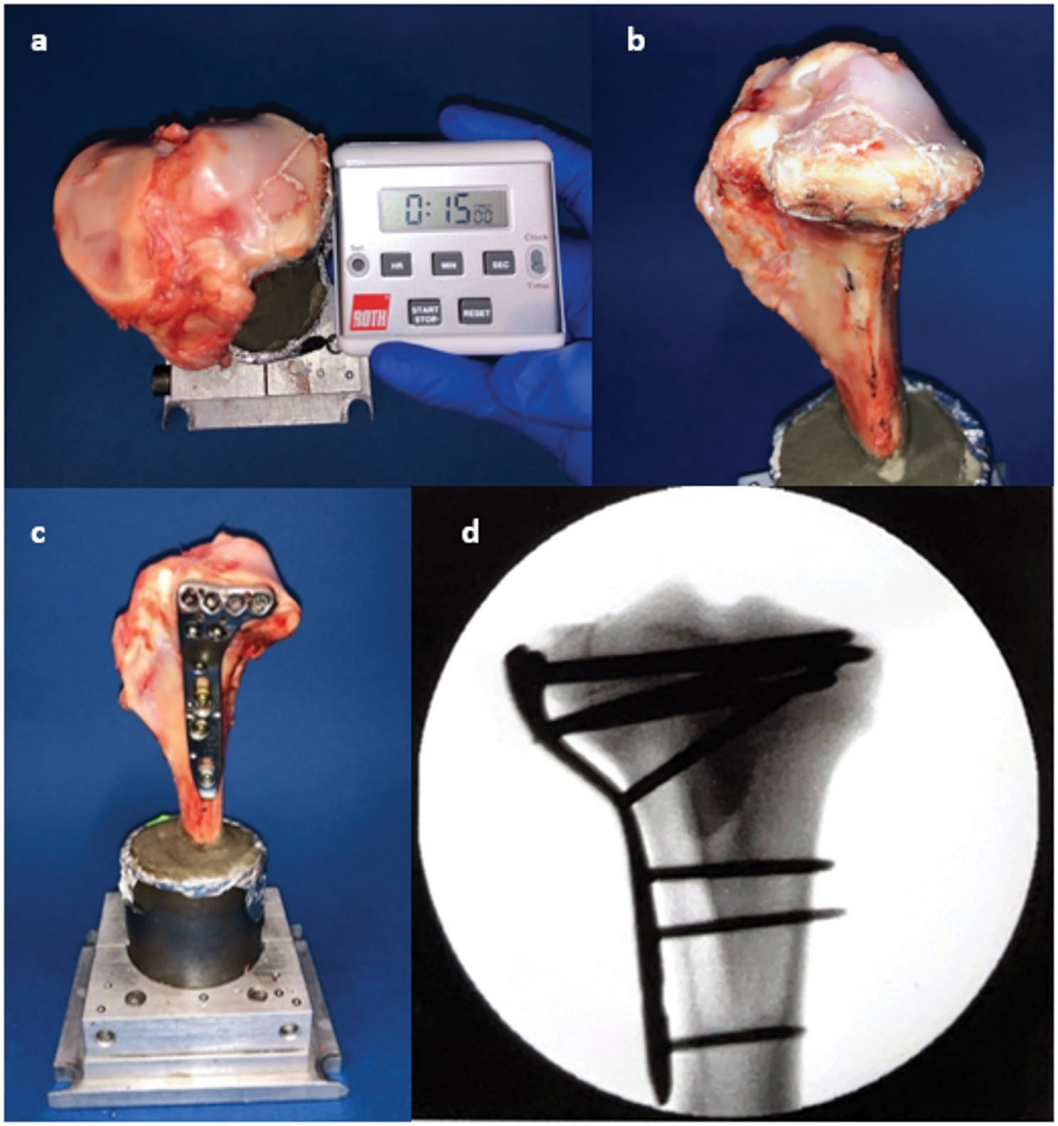
The progress of the operation technique is shown. First, the depression zone was reduced, raised to the anatomical correct level and then the bone cement with a curing phase of 15 min was applied (**a** + **b**). The fracture was then supplied with a combination of cement and plate osteosynthesis from lateral (**c**) and controlled radiographically (**d**)

A total of 27 porcine tibiae were randomly divided into three groups after fracture creation (Table 1). Following anatomical reduction of the fractures, the specimens were stabilized with a plate osteosynthesis in combination with a bone graft substitute to refill the metaphyseal bone defect, which typically remains after reduction of the articular depressed fracture fragment. For the plate osteosynthesis, an actual, clinically often used angle-stable implant, a lateral tibial plate (VA-LCP Proximal Tibial Plate 3.5, DePuy Synthes, Johnson&Johnson Medical GmbH, Norderstedt, Germany), was used. Two different cements were used as bone substitutes, a drillable and simultaneously adhesive magnesium phosphate (MgP) cement and a commercial hydroxyapatite cement, which represented the current standard cement for bone defect filling. The sticky and drillable properties of the MgP cement enabled a new surgical technique, which was used in the first group (A): The complete fracture was reduced and temporarily fixed only by the cement prior to plate osteosynthesis (Fig. 2a, b). In this case, reduction of the split tibial plateau fracture was achieved only by the sticky cement. The drillable properties demonstrated in previous studies allowed easy positioning of the plate and screws afterwards (Fig. 2c) [16, 22]. Specifically, the magnesium phosphate cement was filled into a 5-ml syringe and distributed in the metaphyseal bone defect as well as on the cleavage surfaces to fully maximize its adhesive properties. During the curing time of 15 min at room temperature (22°), compression was applied with a surgical clamp.

In the other two groups, the fracture was first stabilized by plate osteosynthesis after reduction and only then was the bone substitute injected antero-laterally through a gap in the fracture area of the tibia (Table 1). In group B, the same MgP cement from group A was utilized. In group C, a currently clinically used hydroxyapatite cement was applied. All specimens were radiographically verified in two planes for correct fracture reduction and osteosynthesis (Fig. 2d). After fracture stabilization, all specimens were incubated for 24 h at 37. This served to cure the cements while reflecting the load-free phase of bed rest after surgery in the clinical setting. Care was taken to ensure that the bones did not dry out to allow the cements to harden.

**Table 1:** The three different groups with a short description of the operative technique are demonstrated

Group	Description of the Operative Procedure
A	New technique: First fracture reduction only by the sticky MgP cement, then additional plate osteosynthesis
B	Standard procedure by reduction, fixation of the plate osteosynthesis and later application of MgP cement
C	Standard procedure by reduction, fixation of the plate osteosynthesis and later application of a standard hydroxyapatite cement (a-TCP)

### Bone cements

The production of the raw components was carried out at the FMZ, Institute for Functional Materials and Biofabrication at the Julius-Maximilians University of Würzburg. For both cements, 2 kg of the corresponding powder mixture were weighed out per batch and mixed (twice in the case of the magnesium phosphate cement) for one hour in a ploughshare mixer. After transfer to a sintering crucible made of alumina, sintering was carried out at 1400 °C for 5 h with the addition of a bullring to document the temperature. After the sintering process, the phase composition was examined by XRD (X-ray diffraction). The resulting sinter cake was then crushed with a mortar and the powder was sieved until the particles have a size of ≤ 350 μm. Thereafter, 125 g of the powder was placed in each 500 ml zirconia beaker, each containing four zirconia balls of size 30 mm, and then grounded in a planetary ball mill at 200 rpm for one hour. In this way, both Mg_3_(PO4)_2_, MgO and a-TCP were produced.

The magnesium phosphate cement was composed of 7.989 g Mg_3_(PO4)_2_ (ground for 3 h) and 0.583 g MgO combined with 5 ml of 22.5% phytic acid with a powder-to-liquid-ratio (PLR) of 1.714. For the bones used in this study, the amounts were reduced to 1/5, which was sufficient for the fracture surfaces. When mixing, the phytic acid was first added to the MgPO_4_ and mixed until the combination was homogeneous. This was followed by transfer to a glass plate to replenish the MgO and to spread the cement well. This was done for the practical reason that MgO is responsible for curing and thus, from the moment of addition, the application must be done within 5 min. In addition, spreading prevents the mixed cement from clumping.

The hydroxyapatite reference consists of 6 g of a-TCP, which was also sintered for 5 h at 1400 degrees in a large oyte and then pulverized and sieved to a particle size of less than 355 micrograms. To achieve a PLR of 1.714, 3.2 ml of Na_2_HPO_4_ was added. Again, 1.5 g to 0.5 ml was sufficient for fracture supply, with groups B and C requiring less material than the bones of group A.

### Biomechanical test model

**Fig. 3:**
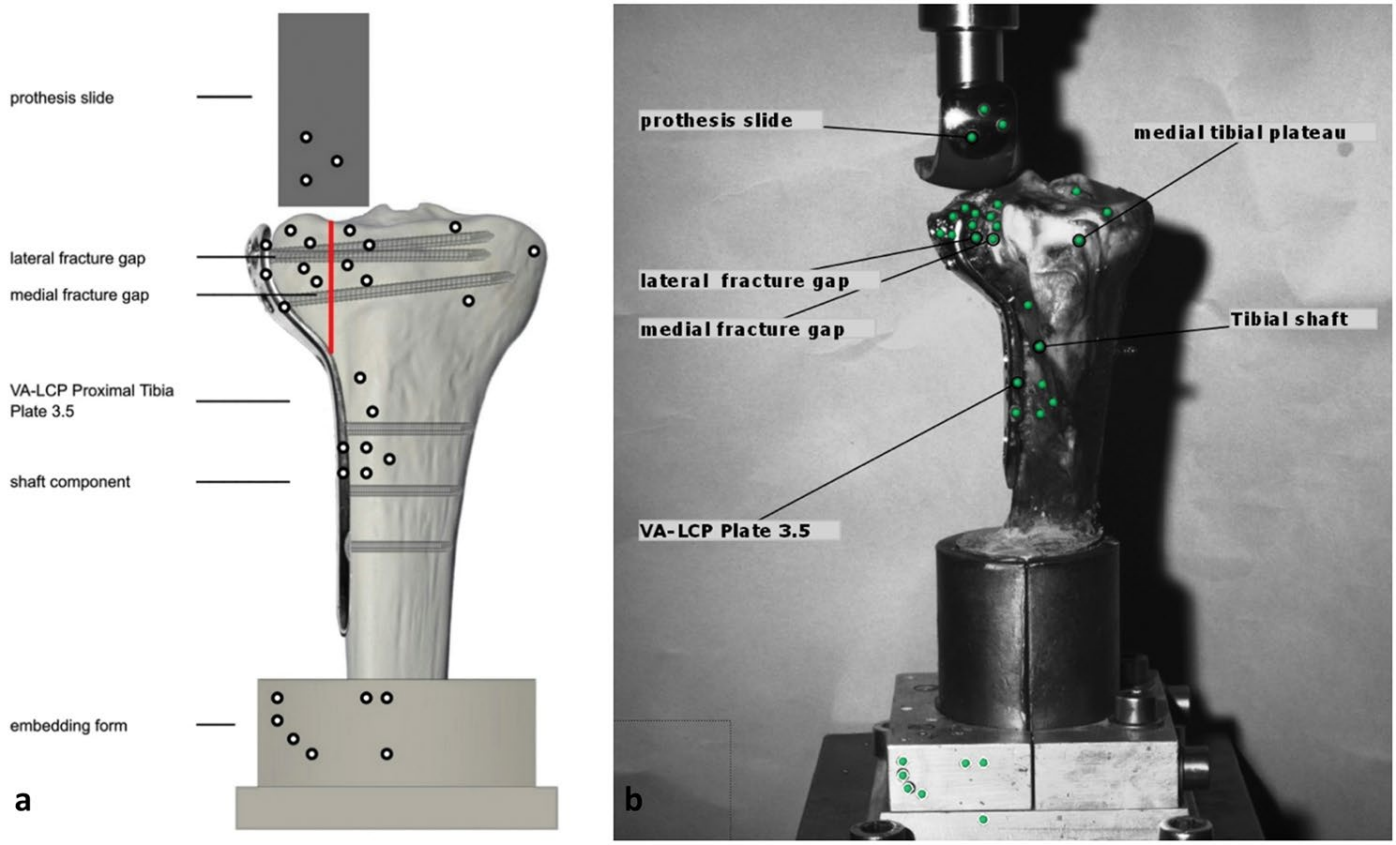
Sketch of the experimental setup and bone with the standardized position of the adhesive dots and element descriptions, the fracture line is visualized with a red line (**a**). Extracted image of a specimen from the optical measuring system with the description of the evaluated elements (**b**)

After fracture stabilization, the bone was prepared with self-adhesive optical marker points beforehand (Fig. 3a) in order to measure the relative change of the position, e.g. displacement, of the elements to each other in the following biomechanical tests. All three groups with the different osteosyntheses methods were cyclically tested at a constant force. The exact load values and number of cycles were determined in a preliminary series of tests: In a pre-testing series with increasing loading of the lateral tibial plateau of specimens (Fig. 3b) stabilized with the standard technique (plate osteosynthesis and filling of the bone defect with hydroxyapatite cement), the loading level with 300 N was determined as the region of interest in loading forces, in which a displacement already occurred after several cycles, but the specimens did not fail immediately. This loading level was a little bit higher than the usual force applied in osteoporotic human bones or their synthetic equivalent, but still reflects the typical partial load bearing requested after surgery (around 250–300 N in the rehabilitation phase). After a holding time of 1 ms at the loading point with 300 N, the load was relieved with 20 N in each case, again at a rate of 25 mm/min.

**Fig. 4:**
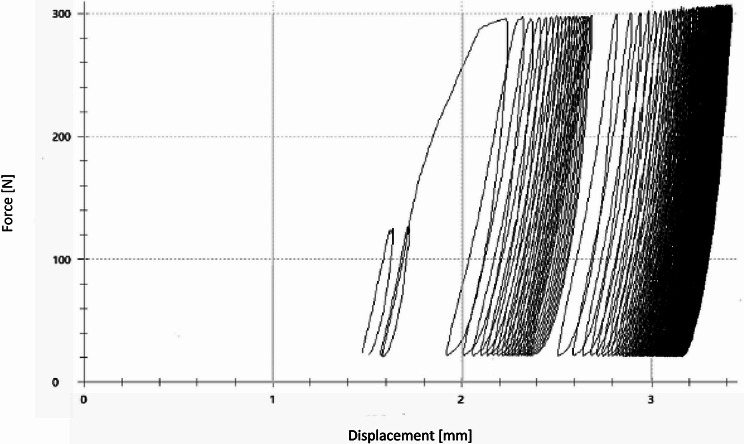
The Load-Displacement curve of the dynamic testing phase with 10 settling and 3,000 measuring cycles is shown. A load from 20 N to 125 N for the settling phase, from 20 N to 300 N was applied on the lateral tiabial plateau

The measuring cycles were preceded by 10 setting cycles performed with a load of 125 N and an unloading of 20 N at a rate of 25 mm/min [10, 21, 23]. According to previous studies, well in agreement with our pre-tests, the number of 3,000 measurement cycles was established (Fig. 4). After the cyclic loading phase, load-to-failure tests were performed applying an increasing axial force at a constant speed of 100 mm/min. This was limited by a maximum depth of depression at 2 cm.

### Parameters of interest

Parameters of interest were total and optical displacement of the lateral plateau [mm] during the cyclic testing phase, stiffness [N/mm], maximum load [N] and normalized maximum load [%] during the load-to-failure tests. The total displacement was recorded by the software of the material testing machine measuring the increasing distance from the start position at cycle one to the final position after 3,000 cycles (Fig. 4). This could be verified with the optical measuring system (ARAMIS 3D Professional, Carl Zeiss GOM Metrology GmbH, Braunschweig, Germany) by measuring the shift of the prothesis sledge into the lateral split fracture during the cyclic loading phase. Therefore, for accurate three-dimensional measurements, three dots were summarized to one element and set in relation to the test setup. The movement of the prothesis slide into the lateral fracture gap was measured based on the mean of three vectors (Fig. 3b).

The load-to-failure-test demonstrated at the beginning of the load-displacement curve a straight line of elastic deformation. The slope of the linear curve was calculated manually for each load-displacement curve and defined as the stiffness.

The peak load of the load-displacement diagram was defined as the maximum load. The initial maximum load of the native bone was determined as the force, which was needed to simulate the split depression fracture. By detecting the initial maximum force, the absolute loads could be divided by the initial maximum load and multiplied by 100 to calculate the normalized maximum load [%]. Interindividual differences of the maximum load were filtered out in this way.

### Statistical analysis

The statistical analysis was performed using SPSS Statistics v.28 (IBM, NY, WI, USA) in cooperation with the Institute of Clinical Epidemiology and Biometry at the University of Würzburg, Germany. The group size was calculated with a G*Power analysis and set in all three groups as *n* = 9 specimens.

The results of this study were first analyzed for normal distribution using the Shapiro-Wilk test. If the data were normally distributed, a one-factor analysis of variance ANOVA was used to determine differences in means. To compare in-between group differences, a Tukey-HSD was added. If the Shapiro-Wilk test did not reveal a normal distribution, the data was analyzed with a non-parametric statistic, a Kruskal-Wallis test. The significance level for all tests was set at *p* < 0.05.

## Results

### Total displacement

**Fig. 5:**
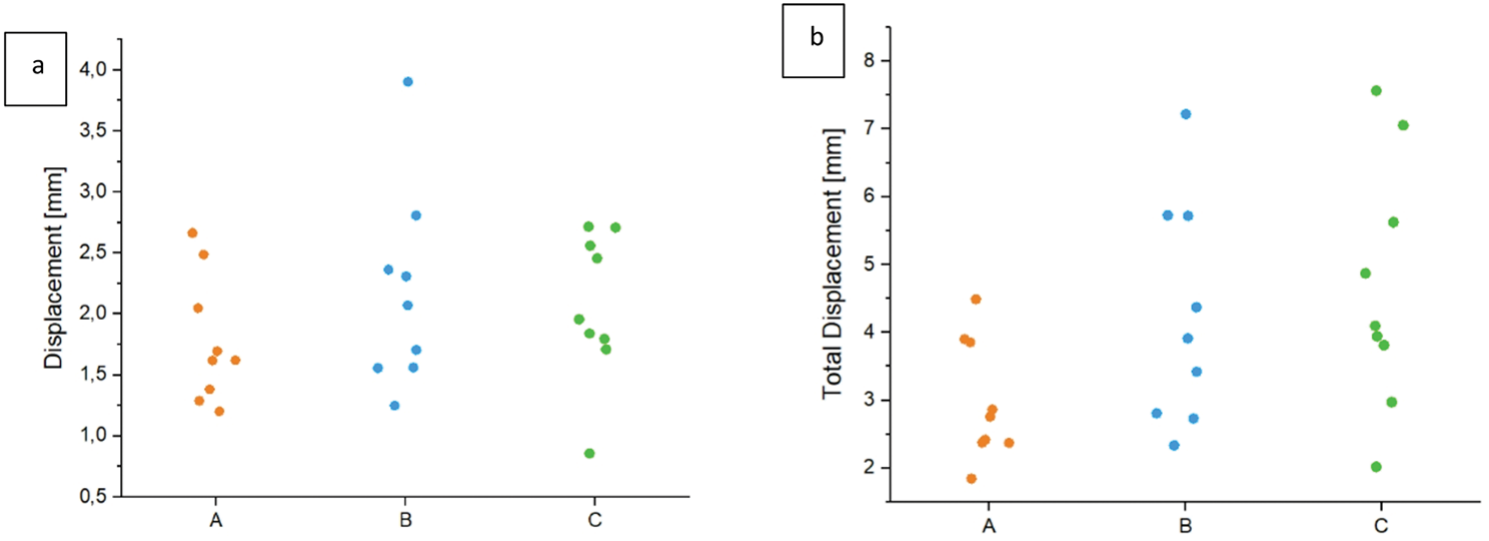
The results (means ± SD) analyzing the displacement in the measuring cycles (**a**) and the total displacement (**b**) recorded by the traverse of the material testing machine are shown. No significant difference between the operation techniques was observed with the lowest values in group A

A normal distribution was found in all measured values. The mean values for the displacement during measuring cycles were 1.78 mm ± 0.52 mm for A, 2.17 mm ± 0.81 mm for B, and 2.07 mm ± 0.61 mm for C. The values of group A tend to be lower than those of groups B and C after 3000 cycles at a constant load of 300 N. However, no significant differences were found (*p* = 0.44) (Fig. 5a). The measured values at the end of the 10 setting cycles and following 3,000 measuring cycles were normally distributed in all groups. The mean values for the total displacement were in group A was 2.98 mm ± 0.89 mm, in group B 4.25 mm ± 1.66 mm and in group C 4.66 mm ± 1.82 mm. Group C exhibited the highest displacement, followed by group B, while group A showed the lowest values measured by the material testing machine. No significant differences could be found in the group comparison (*p* = 0.07) (Fig. 5b).

### Optical displacement of the lateral plateau

**Fig. 6:**
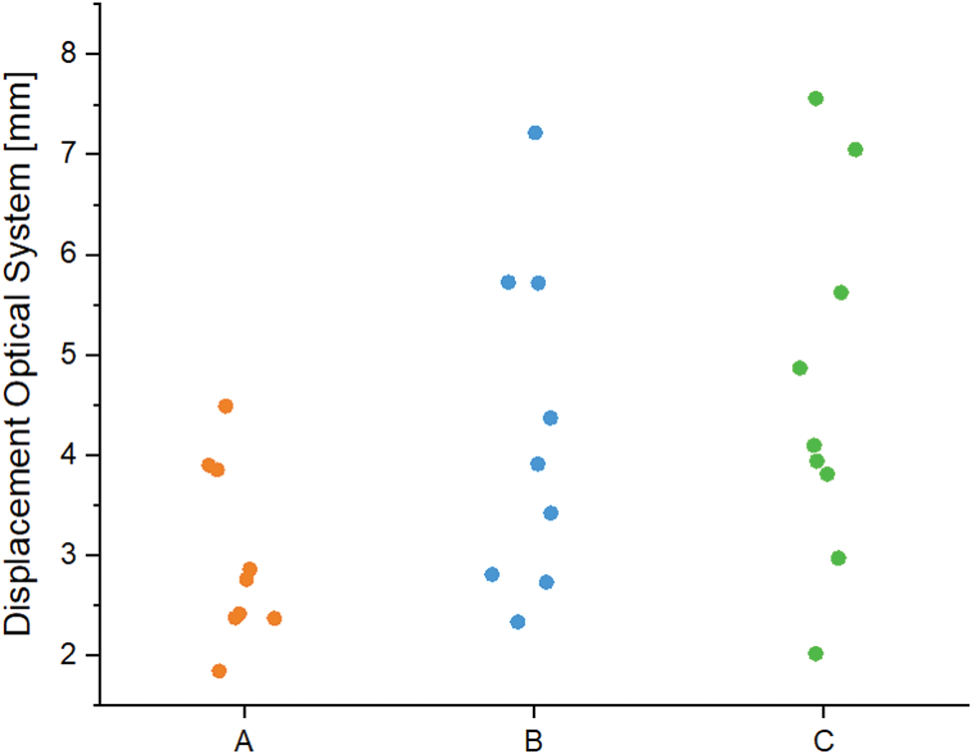
The displacement [mm] detected with the optical system is shown. The comparison of the mean value ± SD demonstrated no significant difference but underlines the trend of a lower displacement in group A

The evaluation of the optical system showed normally distributed results. The change of position of the prothesis slide into the lateral fracture gap was detected and calculated by the mean of three generated vectors in the y-direction. The mean value of the optical displacement is 2.31 ± 1.04 mm for group A, 2.95 ± 0.82 mm for group B and 3.25 ± 1.05 mm for group C. No significant difference was found for the displacement (*p* = 0.15) (Fig. 6). The results showed the previously observed trend of lower values of group A in comparison to B and C.

### Maximum load

**Fig. 7:**
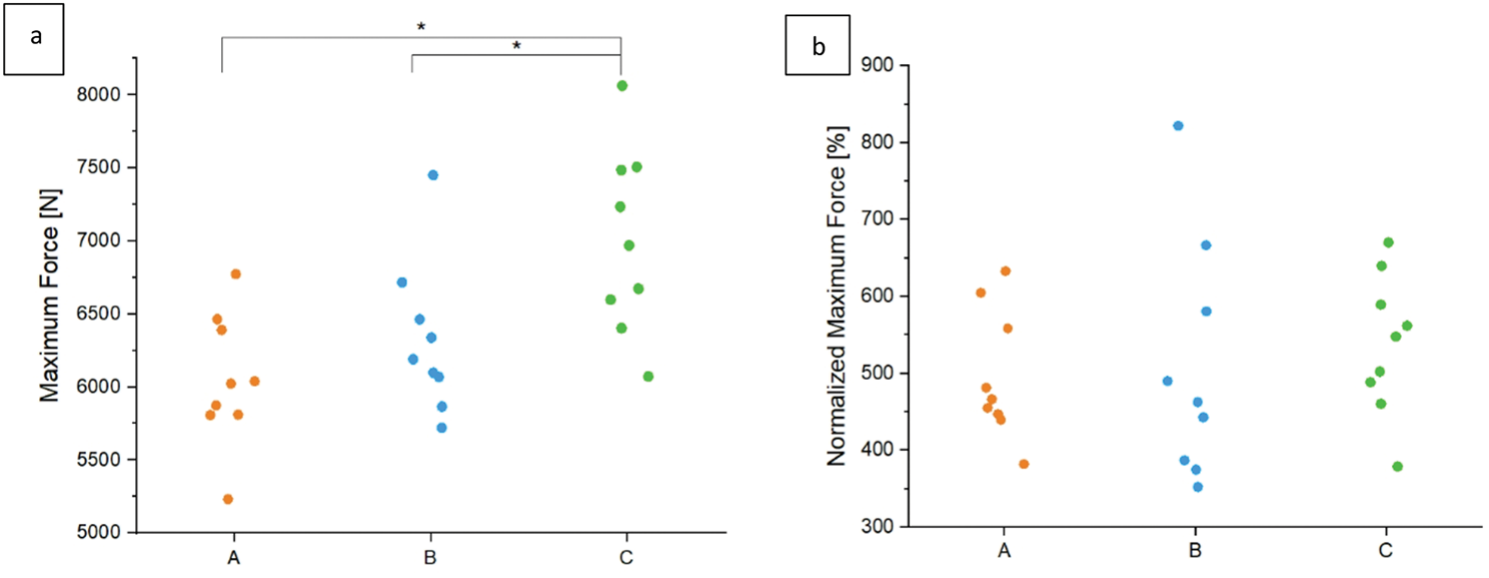
The results of the maximum load (**a**) and the normalized maximum load (**b**) are shown. Means ± SD, significant differences are indicated with * (*p* < 0.05). (**a**) Group C revealed a significant higher maximum load compared to group A (*p* < 0.01) and group B (*p* = 0.04). (**b**) No significant differences could be found for the normalized maximum load, whereas group C showed the highest values

The maximum force of the load-to-failure test Fmax LTF was normally distributed in all groups. For group A the mean value was 6046.05 *N* ± 451.22 N, for group B 6323.51 *N* ± 518.62 N and for group C 7000.61 *N* ± 629.19 N. The difference between A and C was significant with *p* < 0.01 and between B and C with a p-value of 0.04 (Fig. 7a).

All groups demonstrated normally distributed values regarding the parameter of normalized maximum force, measured in percent. The maximum force ratio results in 496.54% ± 83.53% for A, 508.89% ± 154.99% for B, and 537.84% ± 90.91% for C. No significant differences were detected between the groups (*p* = 0.74) (Fig. 7b).

### Stiffness

**Fig. 8:**
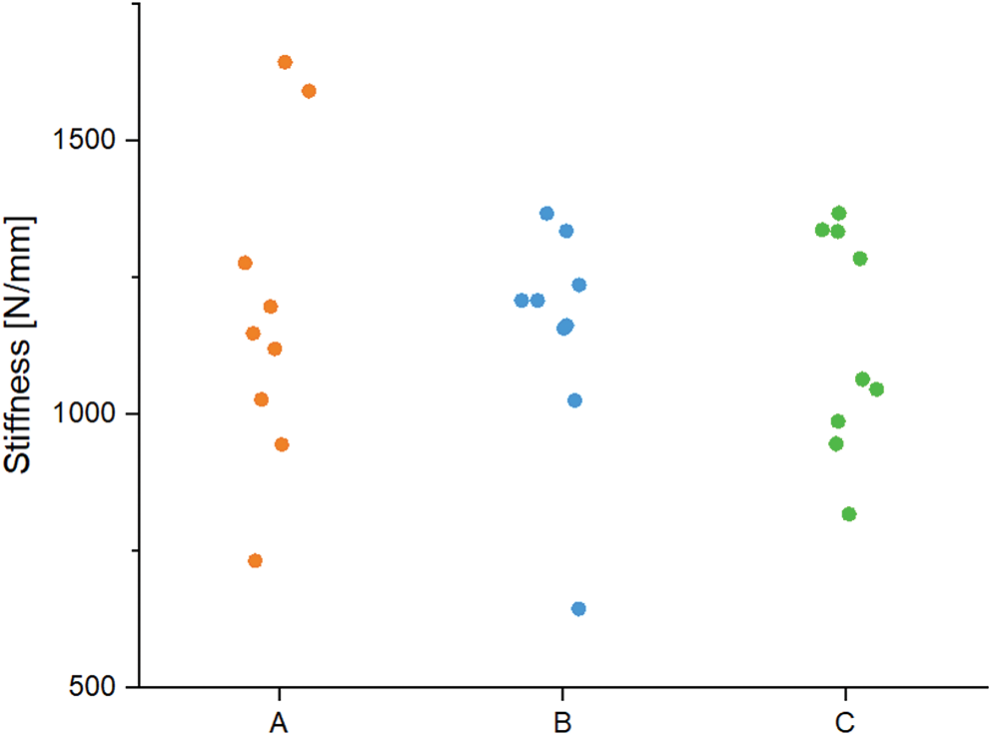
The results of the stiffness are presented. Through all groups the mean values were not significantly different due to the influence of the same plate osteosynthesis used in the different operative techniques

The mean values were as follows: group A 1185.93 N/mm ± 291.07 N/mm, group B 1148.28 N/mm ± 214.12 N/mm and C 1130.84 N/mm ± 202.63 N/mm. There was no significant difference between the groups (*p* = 0.95) (Fig. 8).

## Discussion

Magnesium phosphate cements are promising new bone graft substitutes with clinically relevant properties [14, 24, 25]. In previous studies, the focus was on optimizing the properties regarding handling, processing time, stability and drillability [16, 22]. Likewise, the new cements demonstrated excellent biocompatibility in initial animal studies [13, 15]. An incidental finding in the work with the magnesium phosphate cements, in which phytic acid was used as a liquid component, were adhesive properties in the viscous processing phase of the cements [17]. This fortuitous finding of sticky bone cements suddenly enabled previously unimagined applications: For the first time, in fractures where a bone defect is present, it is possible not only to fill the fracture, but also to bond fracture fragments together simultaneously by adhesive bonding. Only one material is required for this purpose, instead of the usual cement for filling and potentially, a separate bone adhesive, which unfortunately is still not commercially available for clinical use, despite interesting new experimental approaches have been described in literature.: O-phoshpo-L-serine based adhesives revealed promising results as bone and tissue glue [26, 27]. Biomechanically, the bone adhesives showed a high adhesive strength in both studies mentioned, although it should be noted that in both studies a model was selected that only partially reflects the clinical conditions. Mechanical shear tests with bovine bone cubes with prepared bone surface (polishing and drying) are a typical test set-up for a first analysis of bone glues. However, the adhesive characteristics can change considerably when the adhesive comes into contact with body fluids. An interesting approach are screw pullout tests of bonded bone cylinders in human femoral heads. However, human femoral heads, which are generated as a waste product during arthroplasty, exhibit severe changes in bone structure with pronounced subchondral compaction and are therefore only representative of bone bonding in the subchondral region to a limited extent [27, 28]. In this study, we deliberately approached the question of biomechanical testing of a sticky bone cement as an drillable all-rounder in a different way:

Initially, an adequate fracture model had to be established, with a more complex fracture morphology than, for example, simple split fractures. For this project, the fracture must feature components that had both, a part to be glued and a defect to be filled. Therefore, easily generated models by osteotomy were not a consideration. Following *Karunakar et al.*, who created split-depression fractures on human osteoporotic tibial heads [29], a split-depression fracture could also be reproducibly generated on porcine tibial heads. Due to the good bone quality of porcine bones in contrast to osteoporotic human bones, it was very challenging to establish this split-depression model. In combination with predilection sites such as defined holes with rapidly induced forces, it was possible to successfully establish a highly reproducible fracture model. The model is ideally suited for investigating the adhesive and filling properties of a cement. In addition, fractures that require filling of metaphyseal bone defects occur frequently at the tibial plateau. These cements are required to have high primary stability, especially resisting axial loading. In summary, the new model of a split-depression tibial head fracture on porcine bone presented here is an adequate model with high clinical relevance, which is suitable for the question of a new surgical method for filling and bonding fractures with sticky cement.

The, in this study, mentioned new surgical method is the desirable in trauma surgery: reduction and temporary fixation of a complex fracture with only one bone graft material, which allows anatomical reduction and simultaneous filling of a bone defect. It is mentioned at this point that, from our perspective, the use of a bone adhesive in extremity surgery should only be a temporary stabilization and not a definitive treatment, relying solely on the adhesive, as suggested by some authors, who, however, rather refer to other fields of application such as oral and maxillofacial surgery [18, 30]. An application in the highly load-bearing areas of the extremities should be additionally supported by a load carrier, as ensured in this study by plate osteosynthesis. Definitive osteosynthesis of an anatomically reconstructed bone, being held in place by cement, is technically very simple.

Magnesium phosphate cements are used for wastewater treatment due to their pH-regulating and adsorptive properties, and they serve as repair agents in civil engineering due to their rapid stability gain [31]. In addition, they are found as one of the most widely used materials for underfills and as fixings in dentistry, composed of a powder component of magnesium oxide (MgO) or trimagnesium phosphate (Mg_3_PO_4_), also called farringtonite, and a liquid, such as phytic acid, in varying concentrations [31, 32]. An exothermic reaction produces a chelate and thus a stable, complex cement, which - as various works have shown - modified with a phytic acid of 25% and a PLR of around 1.74 g/ml has the best viscosity for application into a fracture gap [16]. Highlighted characteristics, especially in comparison to calcium-based cements, are the higher initial strength and bone affinity as well as a more reliable degradation [33]. Further, temperature development and pH changes also play a decisive role for use under clinical conditions. The latest cements - slightly modified compared to this work - showed values of 27.8–32.5 °C, which can thus be considered as temperatures below a possible risk of heat necrosis or permanent damage. The pH was 6–7.5, corresponding to physiological values at which an optimal function of the cells in relation to their metabolic processes can be assumed [34]. Consequential damage caused by an excessively acidic pH, such as reduced osteoblast function, which would mean bone demineralization and consequently poorer fracture healing, as well as a higher release of proinflammatory cytokines, was not observed. In summary, the magnesium phosphate cements represent a very interesting new group of mineral bone substitute materials, which are characterized by adhesive properties and very good handling in addition to the listed high biocompatibility and primary stability. In this study, the cement used was also convincing due to its adhesive properties as a new all-rounder, with which the above-mentioned new surgical technique of filling fractures simultaneously with bonding is possible for the first time. In addition to enabling the surgical technique, the magnesium phosphate cement group was convincing due to its high biomechanical primary stability with the lowest displacement values and comparable normalized maximum loads.

In the control group, a bone filler was chosen that represents the current standard of injectable cements for bone defects in orthopedic surgery: Tricalcium phosphate cements were first described by Brown and Chow in the 1980s and used at that time first in life in craniofacial defects [33]. It was noticed that a combination of tetra-calcium and dicalcium phosphate resulted in the precipitation of calcium-deficient hydroxyapatite (CDHA) in contrast to brushite and at the same time improved pH-neutral solubility. The final product CDHA is thus most like the actual mineral phase of human bone both crystallographically, chemically and morphologically [35]. Unfortunately, the clinical and basic scientific results of recent years have shown that, contrary to expectations, hydroxyapatite cements do not remodel bone substance even after many years [36]. Therefore, the easily convertible and degradable magnesium phosphate cements represent an interesting alternative, not only because of the above-described properties of drillability and adhesiveness. Furthermore, the results of this study demonstrated that the new surgical technique produces an equally high stability compared to the conventional technique with primary osteosynthesis and only subsequent injection of the cement (for both comparison groups). The results of the new surgical technique demonstrated a trend toward lower displacement and at the same time comparable maximum force, even though without reaching the significance level. Interestingly, the difference in displacement for primary fill and only subsequent screw fixation in the test setup of this study is not significant to the fill after screw fixation as described in a previous study of our research group [10]. However, the fracture morphology tested as well as the test setup of the studies differ so much from each other that, especially due to the different application of force (as here via a sled prosthesis) to the entire lateral tibial plateau, and not only to the area of the depression component of the fracture as in the previous study, there was an uptake of the entire displacement of the lateral plateau. It is likely that the difference in displacement between the groups would have been even more pronounced, if the force had been applied in a less areally distributed manner. Another key difference in the two biomechanical studies is the use of an actual plate design in this presented study as opposed to using only screws [10], whereas the plate even has an improved raft technique with small subchondrally placed screws.

### Limitations

An experimental study always has, of course, limitations: In the here presented model, tibial bones from pigs were used, which biomechanically represent a healthy young bone, but are characterized by the anatomy with a more pronounced flattening of the tibial plateau. Anyway, the fractures on which the tests were performed occur typically in young adults after high energy trauma and not in an old patient cohort with osteoporotic bone quality. Therefore, the best choice with all limitations to use porcine bones for the analysis of our question. Another limitation of an in-vivo study was to investigate only the primary stability and normal physiological aspects of fracture healing were not considered. Beyond, a group size of nine was chosen, calculated based on several preliminary studies of our research group by the Institute of Clinical Epidemiology and Biometry at the University of Würzburg. With a slightly different fracture model and using a different biomechanical test-set up with a prothesis sledge, a model closer to the clinic was selected. However, after looking at the results retrospectively, the variability of the results was higher than expected. Therefore, significant differences could only be shown to a limited extent with a group size of 9. Despite this limitation, the choice of model is very suitable for our research question in order to obtain an initial assessment of the realization of primary anatomical bonding of the fracture and subsequent stabilization by an osteosynthesis.

## Conclusions

This presented study illustrates the use of a bone cement simultaneously as a bone adhesive. The sticky, drillable magnesium phosphate cement is convincing as an all-rounder in the new surgical technique with anatomical reduction of the fracture only by the cement. Furthermore, in the new surgical technique, this cement provided an equal biomechanical stability in the porcine tibial split-depression fracture model compared to the conventional cement in the standard technique.

## Data Availability

Data ist provided within the manuscript.

## Abbreviations

Fig: Figure
Fmax: Force at maximum load (N)
LTF: Load-to-failure
MgP: Magnesium phosphate cement
PLR: Powder-liquid-ratio
Tab: Table
